# Blue Light-Induced Gene Expression Alterations in Cultured Neurons Are the Result of Phototoxic Interactions with Neuronal Culture Media

**DOI:** 10.1523/ENEURO.0386-19.2019

**Published:** 2020-01-02

**Authors:** Corey G. Duke, Katherine E. Savell, Jennifer J. Tuscher, Robert A. Phillips, Jeremy J. Day

**Affiliations:** 1 Department of Neurobiology and Evelyn F. McKnight Brain Institute, University of Alabama at Birmingham, Birmingham, AL 35294; 2 Behavioral Neuroscience Research Branch, Intramural Research Program, National Institute on Drug Abuse, NIH/DHHS, Baltimore, MD 21224

**Keywords:** blue light, immediate early genes, optogenetics, phototoxicity

## Abstract

Blue wavelength light is used as an optical actuator in numerous optogenetic technologies employed in neuronal systems. However, the potential side effects of blue light in neurons has not been thoroughly explored, and recent reports suggest that neuronal exposure to blue light can induce transcriptional alterations *in vitro* and *in vivo*. Here, we examined the effects of blue wavelength light in cultured primary rat cortical cells. Exposure to blue light (470 nm) resulted in upregulation of several immediate early genes (IEGs) traditionally used as markers of neuronal activity, including *Fos* and *Fosb*, but did not alter the expression of circadian clock genes *Bmal1*, *Cry1*, *Cry2*, *Clock*, or *Per2*. IEG expression was increased following 4 h of 5% duty cycle light exposure, and IEG induction was not dependent on light pulse width. Elevated levels of blue light exposure induced a loss of cell viability *in vitro*, suggestive of overt phototoxicity. Induction of IEGs by blue light was maintained in cortical cultures treated with AraC to block glial proliferation, indicating that induction occurred selectively in postmitotic neurons. Importantly, changes in gene expression induced by blue wavelength light were prevented when cultures were maintained in a photoinert media supplemented with a photostable neuronal supplement instead of commonly utilized neuronal culture media and supplements. Together, these findings suggest that light-induced gene expression alterations observed *in vitro* stem from a phototoxic interaction between commonly used media and neurons, and offer a solution to prevent this toxicity when using photoactivatable technology *in vitro*.

## Significance Statement

Technology using blue wavelength light is increasingly used in neuroscience, and recent reports have noted unintended gene expression alterations during light exposure *in vitro*. Here, we identify light-induced gene expression alterations in rat cortical cultures, illustrate that this induction coincides with a loss of cell viability, and show that light induced gene induction is dependent on the culture media used in these experiments. We demonstrate that these unintended effects can be prevented by using phototinert media during to light exposure *in vitro,* opening the door for extended light exposure experiments when using powerful optical techniques in neuronal cultures.

## Introduction

Optically-driven technology has been widely adopted in neuroscientific investigation over the past 15 years (Boyden et al., 2005; Kim et al., 2017), opening new avenues into experimental design by allowing unprecedented spatial and temporal control over neuronal firing, protein signaling, and gene regulation. Blue wavelength light (∼470 nm) is most often used as the actuator of these technologies. For instance, channelrhodopsin (Boyden et al., 2005) is a light-gated ion channel that responds to blue light to allow for experimental control over neuronal firing. Similarly, cryptochrome 2 (Cry2; Kennedy et al., 2010; Konermann et al., 2013; Polstein and Gersbach, 2015) and light-oxygen-sensitive protein (LOV) based systems (Möglich et al., 2009; Dietz et al., 2012; Quejada et al., 2017) use blue light to regulate protein binding and gene expression. Additionally, genetically-encoded calcium sensor technologies to visualize neuronal activity states are becoming more widely used both *in vivo* and *in vitro*, and these sensors often rely on prolonged or repeated blue light exposure (Lin and Schnitzer, 2016; Deo and Lavis, 2018; Wang et al., 2018). Together, these optically-driven technologies provide robust experimental control and have enabled new insights into neuronal functioning in healthy and diseased states. However, increased use of these technologies in neuroscience also warrants a more complete understanding of potential off-target effects of prolonged exposure to blue light.

While the phototoxic effects of both ambient and targeted light on cell viability *in vitro* has been noted for decades (Wang, 1976; Dixit and Cyr, 2003; Carlton et al., 2010), recent reports documenting blue light-induced gene expression alterations both *in vitro* and *in vivo* have emphasized deleterious effects of blue light on cellular function (Marek et al., 2019; Tyssowski and Gray, 2019). Multiple reports have documented robust effects of blue light exposure *in vitro*, including upregulation of genes such as *Fos* (also known as *cFos*) that are often used as markers of neuronal activity but which can also be induced in response to cellular stress (Bahrami and Drabløs, 2016; Marek et al., 2019; Tyssowski and Gray, 2019). Others have noted that cellular phototoxicity is often the result of reactive oxygen species (ROS) generated in culture media during photostimulation, which can be prevented by using a non-light-reactive media instead of the typical media used in neuronal cultures (Stockley et al., 2017). To our knowledge, it has not yet been determined whether the blue light-induced expression alterations of activity-dependent genes observed *in vitro* are the result of a stress response stemming from the culture conditions.

In the present work, we characterized the effects of blue light on gene expression and cell viability *in vitro* using a rat primary neuronal culture model. As recent reports indicate that ROS are generated when culture media is exposed to blue wavelength light (Dixit and Cyr, 2003; Marek et al., 2019), we hypothesized that light-induced alterations in gene expression would be dependent on the neuronal cell culture media used in these experiments. We replicated and extended previous literature by demonstrating that blue light exposure induces multiple immediate early genes (IEGs) in neuronal cultures, and characterized the duration, frequency, and temporal properties of this effect. Notably, we found that replacing cell culture media with a photostable media supplemented with antioxidants prevented blue light-induced gene expression alterations. Together, these experiments provide insight into the mechanism underlying the unwanted “off-target” effects observed when using optically-driven technology, and offer a path forward to achieving a more precise level of experimental control *in vitro*.

## Materials and Methods

### Animals

All experiments were performed in accordance with the University of Alabama at Birmingham Institutional Animal Care and Use Committee. Sprague Dawley timed pregnant rat dams were purchased from Charles River Laboratories. Dams were individually housed until embryonic day (E)18 for cell culture harvest in an AAALAC-approved animal care facility on a 12/12 h light/dark cycle with *ad libitum* food and water.

### Cortical cell cultures

Primary rat cortical cultures were generated from E18 rat cortical tissue, as described previously (Day et al., 2013; Savell et al., 2016, 2019). Briefly, cell culture plates (Denville Scientific Inc.) were coated overnight with poly-L-lysine (Sigma-Aldrich; 50 μg/ml) and rinsed with diH_2_O. Dissected cortical tissue was incubated with papain (Worthington LK003178) for 25 min at 37°C. After rinsing in complete Neurobasal media [Neurobasal Medium (Gibco; #21103049), supplemented with B27 (Gibco; #17504044, 1× concentration) and L-glutamine (Gibco; # 25030149, 0.5mM)], a single-cell suspension was prepared by sequential trituration through large to small fire-polished Pasteur pipettes and filtered through a 100-μm cell strainer (Fisher Scientific). Cells were pelleted, re-suspended in fresh media, counted, and seeded to a density of 12, 000 cells per well on 24-well culture plates (65,000 cells/cm^2^). Cells were grown in complete Neurobasal media for 11 d *in vitro* (DIV) in a humidified CO_2_ (5%) incubator at 37°C with half media changes at DIV1 and DIV5. On DIV10, cells received either a half or full change to complete Neurobasal media, or complete NEUMO media [Neumo Media (Cell Guidance Systems; M07-500) supplemented with SOS (Cell Guidance Systems; M09-50, 1× concentration) and Glutamax (Thermo Fisher; 35050061, 1× concentration)], as indicated above. In experiments comparing complete Neurobasal media to complete NEUMO media, Glutamax at a 1× concentration was used in place of L-glutamine for the complete Neurobasal media DIV10 media change, so that the effects of SOS/NEUMO and Neurobasal/B27 could be compared directly. To block glial proliferation, β-D-arabinofuranoside hydrochloride (AraC; Sigma-Aldrich) was added to complete Neurobasal media on DIV4 to achieve a final concentration of 5 μM, as previously described (Henderson et al., 2019). These culture wells received half media changes on DIV1, DIV7, and a full media change on DIV10 with complete Neurobasal media before light exposure on DIV11. Control wells received the same media changes with no AraC present on the DIV4 media change.

### Illumination

A custom built 12 LED array was used to illuminate cells, as previously described (Polstein and Gersbach, 2014). Three series of four blue LEDs [Luxeon Rebel Blue (470 nm) LEDs; SP-05-B4] regulated by a 700-mA BuckPuck (Luxeon STAR) were mounted and soldered onto a rectangular grid circuit board (Radioshack) and positioned inside a plastic enclosure (Radioshack) beneath transparent Plexiglas (2 mm thick). Primary cortical culture plates were positioned atop this enclosure and illuminated from below. Irradiance was determined through an empty culture plate placed atop the light box at six positions without a foil wrapping and at two positions while encased in foil using a spectrophotometer (Spectrascan PR-670; Photo Research). Irradiance ranged from 0.40 mW/cm^2^ in the corner position (0.42 mW/cm^2^ while under foil), to 0.84 mW/cm^2^ in the center (0.91 mW/cm^2^ while under foil). An Arduino Uno was used to control LED arrays, delivering light in 1-s pulses at the frequencies required to achieve specific duty cycles. In all experiments, duty cycle percentage was defined as light on time/total time × 100. Aluminum foil was placed on top of the culture dish and enclosure during light delivery. No-light control culture plates were placed atop an identical LED enclosure and wrapped in foil. All handling of culture plates was performed under red light conditions after DIV5.

### RNA extraction and RT-qPCR

Total RNA was extracted (RNAeasy kit, QIAGEN) and reverse-transcribed (iScript cDNA Synthesis kit, Bio-Rad) following the manufacturers’ instructions. cDNA was subject to RT-qPCR for genes of interest in duplicate using a CFX96 real-time PCR system (Bio-Rad) at 95°C for 3 min, followed by 40 cycles of 95°C for 10 s and 58°C for 30 s, followed by real-time melt analysis to verify product specificity, as described previously (Savell et al., 2016, 2019). *Gapdh* was used for normalization via the ΔΔCt method (Livak and Schmittgen, 2001). A list of PCR primer sequences is provided in Table 1.

**Table 1.** RT-qPCR primer sets

Gene	Forward primer	Reverse primer
*Gapdh*	ACCTTTGATGCTGGGGCTGGC	GGGCTGAGTTGGGATGGGGACT
*Fos*	CAGCCTTTCCTACTACCATTCC	ACAGATCTGCGCAAAAGTCC
*Egr1*	TCCTCAAGGGGAGCCGAGCG	GGTGATGGGAGGCAACCGGG
*Fosb*	TGCAGCTAAATGCAGAAACC	CTCTTCGAGCTGATCCGTTT
*Arc*	GCTGAAGCAGCAGACCTGA	TTCACTGGTATGAATCACTGCT
*Bdnf IV*	GCTGCCTTGATGTTTACTTTGA	GCAACCGAAGTATGAAATAACC
*Per2*	CACCCTGAAAAGAAAGTGCGA	CAACGCCAAGGAGCTCAAGT
*Cry1*	AAGTCATCGTGCGCATTTCA	TCATCATGGTCGTCGGACAGA
*Cry2*	GGATAAGCACTTGGAACGGAA	ACAAGTCCCACAGGCGGT
*Clock*	TCTCTTCCAAACCAGACGCC	TGCGGCATACTGGATGGAAT
*Bmal1*	CCGATGACGAACTGAAACACCT	TGCAGTGTCCGAGGAAGATAGC

RT-qPCR primer sets used in the experiments detailed in this article.

### Calcein AM viability assay

Cell viability was assessed using a Calcein AM Cell Viability Assay kit (Trevigen; 4892-010-K) according to manufacturer’s instructions for adherent cells. Briefly, cell culture media was removed followed by a wash with 400 μl of Calcein AM DW buffer; 200 μl of Calcein AM DW buffer and 200 μl of Calcein AM Working solution were then added to the culture well and allowed to incubate at 37°C in a humidified CO_2_ (5%) incubator for 30 min. Culture well florescence was then assessed under 470-nm excitation in a standard plate imager (Azure Biosystems c600), and quantified in ImageJ by taking the background subtracted mean pixel value of identical regions of interest areas encompassing individual culture wells. Background was calculated for subtraction by taking the mean pixel value of two regions above and below the cell culture plate.

### Immunocytochemistry

Immunostaining to assess the cell-type composition of the primary cortical cultures was performed as described previously (Savell et al., 2016). After removal of neuronal culture media, cells were washed with PBS and incubated at room temperature for 20 min in freshly prepared 4% paraformaldehyde in PBS. After fixation, cells were washed twice with PBS and neuronal membranes were permeabilized with PBS containing 0.25% Triton X-100 for 15 min at room temperature. Cells were then washed three times in PBS, blocked for 1 h [10% Thermo Blocker bovine serum albumin (BSA) #37525, 0.05% Tween 20, and 300 mM glycine in PBS] and co-incubated with Anti-NeuN Antibody, clone A60, Alexa Fluor 555 conjugate (1:100 in PBS with 10% Thermo Blocker BSA Millipore Sigma catalog #MAB377A5, RRID: AB_2814948) and anti-glial fibrillary acidic protein antibody, clone GA5, Alexa Fluor 488 (1:250 in PBS with 10% Thermo Blocker BSA, Millipore Sigma catalog #MAB3402X, RRID: AB_11210273) overnight at 4°C. Cells were then washed twice with PBS containing 0.25% Triton X-100, followed by a final wash with PBS for 10 min. Slide covers slips with Prolong Gold anti-fade medium (Invitrogen) containing 4,6-diamidino-2-phenylindole (DAPI) stain were placed atop the culture wells. A Nikon TiS inverted fluorescent microscope was used to capture 10× magnification (1,888-mm^2^ field of view) images from six wells (two images/well) from a 24-well culture plate. Total number of NeuN and GFAP-positive cells were quantified for each image captured using Cell Counter in ImageJ v2.0.0. Values for each cell population are expressed as a percentage of the total combined (GFAP+NeuN) number of cells.

### Statistical analysis

Transcriptional differences from RT-qPCR experiments were compared with either an unpaired *t* test or one-way ANOVA with Dunnett’s or Tukey’s *post hoc* tests where appropriate. Statistical significance was designated at α = 0.05 for all analyses. Statistical and graphical analyses were performed with Prism software (GraphPad). Statistical assumptions (e.g., normality and homogeneity for parametric tests) were formally tested and examined via boxplots.

### Data availability

All relevant data that support the findings of this study are available by request from the corresponding author.

## Results

### Blue light induces IEG expression in primary cortical cultures

**Figure 1.**
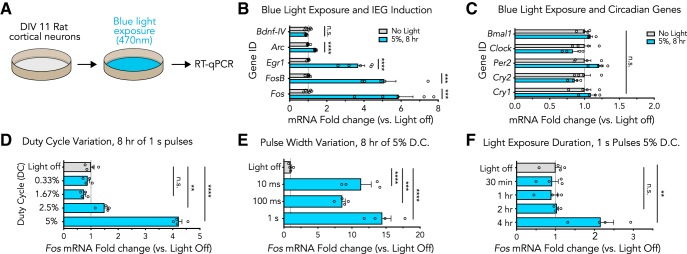
Blue light induces IEG expression in primary cortical cultures. ***A***, Illustration of the experimental design. Primary rat cortical cultures were placed on top of a light box and exposed to blue (470 nm) light before measurement of gene expression with RT-qPCR. ***B***, Blue light induces gene expression alterations at multiple IEGs (*n* = 5, unpaired *t* test; *Fos t*_(8)_ = 6.301, *p* = 0.0002; *Fosb t*_(8)_ = 6.384, *p* = 0.0002; *Egr1 t*_(8)_ = 7.613, *p* < 0.0001; *Arc t*_(8)_ = 10.54, *p* < 0.0001; *Bdnf-IV t*_(8)_ = 1.563, *p* = 0.1566). ***C***, Circadian rhythm genes were not altered by this blue light exposure (*n* = 4, unpaired *t* test; *Bmal1 t*_(6)_ = 1.772, *p* = 0.1268; *Clock t*_(6)_ = 1.499, *p* = 0.1845 *Per2 t*_(6)_ = 1.910, *p* = 0.1048; *Cry2 t*_(6)_ = 1.491, *p* = 0.1865; *Cry1 t*_(6)_ = 0.7978, *p* = 0.4554). ***D***, *Fos* gene expression alterations are dependent on the amount of light exposure received (*n* = 4, one-way ANOVA; *F*_(4,15)_ = 215.1, *p* < 0.0001). ***E***, Gene induction is not dependent on pulse width when duty cycle is held constant (*n* = 4, one-way ANOVA; *F*_(3,12)_ = 32.96, *p* < 0.0001). ***F***, Gene expression is altered as early as 4 h after light exposure (*n* = 4, one-way ANOVA; *F*_(4,15)_ = 9.075, *p* = 0.0006). All data are expressed as mean ± SEM. Individual comparisons, **p < 0.01, ***p < 0.001, ****p < 0.0001, n.s. = not significant. D.C. = duty cycle.

To investigate the effects of blue light exposure on gene expression *in vitro*, we exposed DIV11 primary cortical cultures to 470-nm light and monitored gene expression with reverse transcription quantitative PCR (RT-qPCR; Fig. 1). Cortical cells cultured in standard media conditions (complete Neurobasal supplemented with B27) were placed on top of a blue LED array light box (Polstein and Gersbach, 2014) inside of a standard cell culture incubator. Pulsed 470-nm light was delivered across seven duty cycle conditions for 0.5–8 h, followed by RT-qPCR to compare gene expression of light-exposed plates to control plates that were not exposed to light (Fig. 1*A*). First, neuronal cultures were exposed to 5% duty cycle (1-s pulses every 19 s) light for 8 h, and RNA was extracted to examine the effects of blue light exposure on IEG expression. RT-qPCR revealed significant induction of *Fos*, *Fosb*, *Egr1*, and *Arc* mRNA, but not mRNA arising from *Bdnf-IV* (Fig. 1*B*). To determine whether blue light exposure had an effect on the circadian clock, expression of circadian rhythm genes *Bmal1*, *Clock*, *Per2*, *Cry2*, and *Cry1* was measured under same light exposure conditions. In contrast to robust changes in IEGs, no significant light-induced changes were documented at these key circadian rhythm genes (Fig. 1*C*).

Optogenetic methods often rely on precise programs of light stimulation. Therefore, we sought to understand whether the duty cycle, pulse width, or duration of blue light influenced the induction of IEGs, using *Fos* mRNA as a representative marker. First, we varied the duty cycle to determine whether IEG induction scaled with increased light exposure. *Fos* mRNA was significantly induced at duty cycles of 5% and 2.5%, but not at 1.67% or 0.33% (Fig. 1*D*). Next, while maintaining 5% duty cycle light exposure for 8 h, we varied the light pulse width to determine whether the same total light exposure at different frequencies would impact the induction of *Fos* mRNA. All light pulse variations induced expression of *Fos* mRNA to similar levels, indicating that this effect was not dependent on pulse frequency (Fig. 1*E*). Finally, we sought to identify the duration of light exposure necessary to induce *Fos* mRNA by varying the overall length of light exposure. We detected differences in *Fos* mRNA at 4 h after light exposure began, but not at earlier timepoints (Fig. 1*F*). Taken together, these results demonstrate that blue wavelength light can alter gene expression in cortical cultures at relatively low duty cycles, that this effect is insensitive to specific exposure frequencies, and that longer exposure times were required to observe transcriptional responses at a 5% duty cycle.

### Blue light is phototoxic to primary cortical cultures

**Figure 2.**
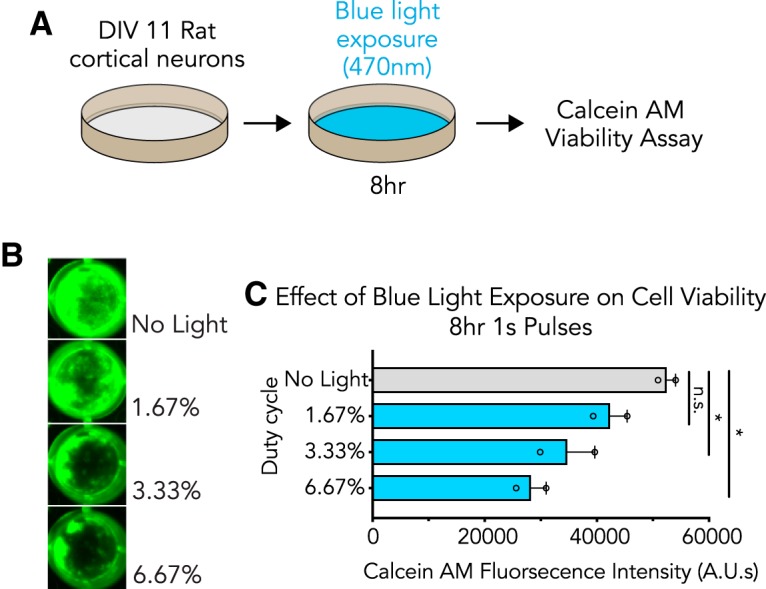
Blue light is phototoxic to primary cortical cultures. ***A***, Illustration of the experimental design. Primary rat cortical cultures were exposed to blue wavelength light before cell viability was assessed with a Calcein AM assay. ***B***, Blue light causes a loss in cell viability with increased light exposure. ***C***, Quantified effects of blue light exposure on cell viability at different duty cycles (*n* = 2, one-way ANOVA; *F*_(3,4)_ = 10.20, *p* = 0.0241). All data are expressed as mean ± SEM. Individual comparisons, *p < 0.05, n.s. = not significant.

To understand whether light-induced gene expression alterations corresponded with changes in cell health, we next examined the effects of blue light exposure on cell viability (Fig. 2). Primary cortical cultures were exposed to blue light (470 nm) for 8 h (at 1.67%, 3.33%, and 6.67% duty cycles) before assessing cell health using fluorescence measurements in a Calcein AM viability assay in which decreased fluorescence marks a loss in cell viability (Fig. 2*A*,*B*). We observed decreased fluorescence intensity at both 3.33% and 6.67% light exposure as compared to a no-light control, indicative of cell death at these duty cycles (Fig. 2*C*). These findings suggest that cellular health is significantly impacted during sustained light exposure, correlating IEG induction with a loss in cellular viability.

### Glia-depleted cortical cultures maintain blue light-induced gene expression alterations

**Figure 3.**
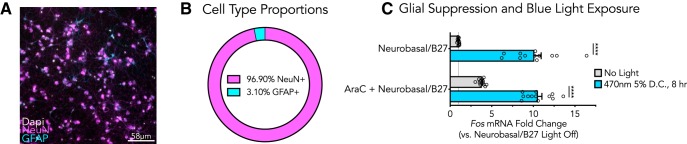
Glia depleted cortical cultures maintain blue light-induced alterations in *Fos* mRNA expression. ***A***, Immunocytochemistry for NeuN and GFAP in primary rat cortical cultures. ***B***, Quantification of NeuN+ and GFAP+ cells revealed that 96.9% of positively stained cells were NeuN+ across six culture wells. ***C***, Depletion of glial cells using AraC (5 μM) supplemented culture media did not prevent blue light-induced gene expression changes (*n* = 12, unpaired *t* test; *Neurobasal/B27 t*_(22)_ = 11.19, *p* ≤ 0.000001; *AraC + Neurobasal/B27 t*_(22)_ = 13.82, *p* ≤ 0.000001). All data are expressed as mean ± SEM. Individual comparisons, ****p < 0.0001. D.C. = duty cycle.

Next, we investigated whether these alterations were neuron specific, as E18 rat primary cortical cultures often contain trace amounts of glial growth (Fig. 3). Immunostaining of GFAP confirmed that glial cells were present in these primary cortical cultures (Fig 3*A*), but in small numbers relative to NeuN+ neuronal cells (3.10% of positively stained cells were GFAP+ against NeuN staining across six culture wells, Fig. 3*B*). To determine whether the blue light-induced gene expression response was dependent on the presence of proliferating glial cells, cytosine arabinoside (AraC, an inhibitor of DNA synthesis) was applied to deplete the cultures of dividing glial cells before light exposure (Fig. 3*C*). The cultures were then exposed to blue light for 8 h at a 5% duty cycle and *Fos* gene expression was monitored. *Fos* mRNA was significantly increased in the light exposure groups relative to light-off controls to similar levels in both AraC treated wells and in control wells receiving no AraC treatment, suggesting that these blue light-induced effects are not dependent on glial presence. Together, these results demonstrate that cortical cell cultures used here contain only a small fraction of glial cells and demonstrate that glia are not required for light-induced transcriptional alterations.

### Photoinert media protects cortical cultures from blue light-induced gene expression alterations

**Figure 4.**
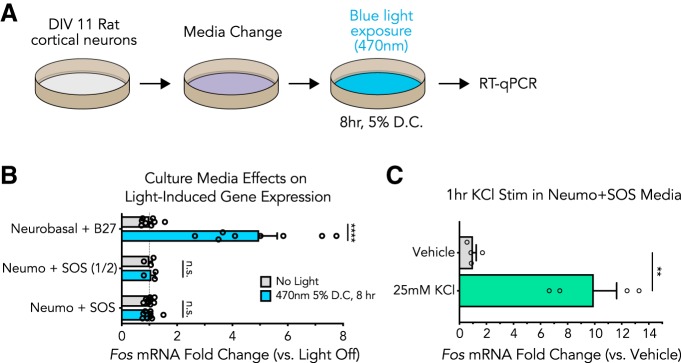
Photoinert media protects cortical cultures from blue light-induced gene expression alterations. ***A***, Illustration of the experimental design. Primary rat cortical cultures were exposed to blue wavelength light 12 h following a media change and then gene expression was assessed by RT-qPCR. ***B***, Blue light exposure does not induce *Fos* mRNA changes in photoprotective culture media, even if only a half media change is performed [*n* = 3–9, unpaired *t* test; Neurobasal *t*_(14)_ = 6.012, *p* = 0.000032; Neumo (1/2) *t*_(4)_ = 0.4099, *p* = 0.708249; Neumo (full) *t*_(16)_ = 0.02414, *p* = 0.981036]. ***C***, *Fos* mRNA can be induced by a 1-h 25mM KCl stimulation in photoprotective media, indicating that the cultures are still capable of induced gene expression alterations (*n* = 4, unpaired *t* test, two-tailed; *t*_(6)_ = 5.221, *p* = 0.0020). All data are expressed as mean ± SEM. Individual comparisons, **p < 0.01, ****p < 0.0001, n.s. = not significant. D.C. = duty cycle.

Recent reports suggest light-induced cell viability losses can be overcome with photoinert media (Stockley et al., 2017), but it remains unclear whether light-induced gene expression effects are also dependent on the culture media used in these experiments. To examine the contributions of culture media to light-induced gene expression changes, we explored the effects of light exposure in neurons cultured in photoinert media (Fig. 4). Culture media was replaced 12 h before light exposure with a full or half media change to either Neumo + SOS or Neurobasal + B27 before blue light exposure (8 h at 5% duty cycle; Fig. 4*A*). Interestingly, both a full and a half media change to photoinert media completely blocked light-induced *Fos* mRNA increases observed when using standard neuronal culture media (Fig. 4*B*). To confirm that neurons cultured in photoinert media remained physiologically capable of *Fos* gene induction, we depolarized neurons for 1 h with potassium chloride (KCl, 25mM) stimulation in this media and observed significant upregulation of *Fos* mRNA (Fig. 4*C*). Taken together, these results suggest that light-induced upregulation of IEGs in cultured neuron experiments are the result of an interaction with light and culture media, not the result of a direct cellular response to light.

## Discussion

The increased adoption of optical techniques requiring prolonged light exposure in neuroscience highlights a pressing need to both characterize and overcome any off-target effects due to light exposure alone. To better understand the effects of blue light exposure in cultured neurons, we exposed primary cortical cultures to blue wavelength light and monitored gene expression alterations and cell viability changes. We observed significant elevation of multiple IEGs in primary cultures in response to blue light, noting that this induction is dependent on the amount of light delivered, and that alterations occur after 4 h of photostimulation or more. The IEGs we characterized are downstream of the ERK/MAPK pathways and upregulated in response to robust synaptic activation during long-term plasticity induction (Sheng and Greenberg, 1990; West and Greenberg, 2011; Chung, 2015). However, these genes are also triggered in response to cellular stress, including exposure to reactive oxygen species at timescales consistent with those used here (Janssen et al., 1997; Hughes et al., 1999; Chaum et al., 2009; Bahrami and Drabløs, 2016). In contrast, we observed no alterations in expression of circadian rhythm machinery genes, suggesting that this IEG response was not due to light-induced alterations of the circadian cycle. The role of IEG family members in survival and programmed cell death are well known, with IEG induction often preceding and playing critical functions in apoptosis programs (Smeyne et al., 1993; Haby et al., 1994; Morris, 1995; Janssen et al., 1997; Ameyar et al., 2003; Gazon et al., 2017). To determine whether this transcriptional response is indicative of cellular stress, we examined cell viability across increasing light exposures, demonstrating a decrease in cell viability with increasing amounts of blue light. These results suggest that the gene expression changes we observed following blue light exposure are associated with a cellular stress response.

Previous reports have found that culture media and its supplements can react with light to generate ROS, and recent efforts to overcome this have resulted in the generation of photostable culture media which prevents a decay in cell health during sustained light exposure (Wang, 1976; Dixit and Cyr, 2003; Stockley et al., 2017; Marek et al., 2019). Importantly, we report that blue light-induced alterations in IEGs such as *Fos* are prevented when neuronal culture media is transitioned to photostable solution supplemented with antioxidants before light exposure. While in this photostable media, neurons maintain their ability to elicit IEG induction following strong depolarization, indicating that the light-induced gene response is dependent on culture media and can be readily overcome.

With the rapid and widespread adoption of light-inducible technologies in neurobiology (Rost et al., 2017), these results provide a path forward when using these techniques *in vitro*. Recent reports have documented light-induced gene expression alterations of *Fos in vivo* (Villaruel et al., 2018), which may be the result of a similar stress response from poor heat dissipation during extended exposure times *in vivo* (Owen et al., 2019). In sum, our study highlights the importance of experimental design when using photoactivatable and imaging technologies. Specifically, these results highlight the necessity of including a light exposure only control group when adapting these promising techniques to particular experimental conditions, and the utilization of photostable culture media wherever possible. Improving experimental precision and accuracy is of high priority given the remarkable experimental control and power these techniques provide. Together, the approach outlined here offers an easily implementable solution for the integration of photoactivatable technologies to neuroscientific inquiry *in vitro* that mitigates experimental confounds due to phototoxicity.

## Synthesis

Reviewing Editor: Juan Burrone, King's College London Decisions are customarily a result of the Reviewing Editor and the peer reviewers coming together and discussing their recommendations until a consensus is reached. When revisions are invited, a fact-based synthesis statement explaining their decision and outlining what is needed to prepare a revision will be listed below. The following reviewer(s) agreed to reveal their identity: Matthew Grubb.

Reviewer 1

This study clearly demonstrates biological dose-dependent effects of illuminating neurons/cells with blue light. Including both analysis of IEGs and circadian genes is elegant and rules out direct light-dependent effects on 'light-sensitive' genes. Going further to demonstrate that simply changing composition of the medium is sufficient to alleviate the light-dependent effects will be extremely helpful for many researchers using optogenetic tools. It has long been known that light (particularly UV/violet/blue) has direct effects on various parameters of cell viability. These older studies are acknowledged and this study goes further than just a new demonstration of the 'problem' but advances the field by pointing out the solution, to change the cell culture medium composition.

The next question (outside the scope of this paper) will be to determine if the in vivo effects of light are more similar to the effects in vitro in classical medium or to the Neumo medium.

I have two main comments that should be addressed before publication:

1) In the methods please describe the exact composition of the media, i.e. how much B27/glutammine, SOS/glutamax were added, was there insulin or any other supplements? To be able to follow this protocol these should be precisely given. The manufacturer gives some guidelines but exact composition might change the results.

2) What is the light intensity at the cultures? Duty cycle doesn't tell much about the light dose without the power (in Watts per unit area or Joules). How intensity was measured/calibrated is also necessary for anyone to replicate these experiments or to ensure they are working within the tested 'safe' light range.

Reviewer 2

This is a well-written manuscript presenting some interesting and extremely useful data on gene expression changes induced by blue light stimulation in neuronal cell culture, and ways to mitigate such unwanted effects. The data and analyses appear very sound. I have only one major comment and several much smaller concerns, outlined below:

Major comment:

The authors describe their cell cultures as 'neuronal cell cultures' and refer to their results as pertaining specifically to neurons (e.g. first subtitle of Results section). However, there are no data showing that their cultures are purely neuronal. If the cultures contain at least some glial cells, there is the possibility that many of the main results of the paper could be explained by blue light-induced cell death acting preferentially on glia. The effects on gene expression (e.g. Fig1b) are coincident with significant effects on cell survival (Fig2). So, if blue light killed glia more effectively than neurons, this could theoretically account for increases in neuronal gene expression (e.g. Fos; i.e. gene expression per cell does not increase, but the proportion of cells expressing the gene does). Sure, expression of some genes is not affected by blue light stimulation, but those genes are not exclusively neuronal (https://www.ncbi.nlm.nih.gov/pmc/articles/PMC5037500/ ; https://www.ncbi.nlm.nih.gov/pubmed/18649393), so might not be affected by changes in the glial:neuronal ratio induced by blue light stimulation. In order to be sure that blue light is indeed directly increasing gene expression in neurons, rather than producing illusory gene expression changes due to preferential glial cell death, the authors should 1) demonstrate that their cultures are (almost) exclusively neuronal; and/or 2) show examples of neuron- or glial-specific genes whose expression is not altered by blue light stimulation at 5% duty cycle for 8h; and/or 3) show that cell death induced by blue light stimulation does not preferentially act on glia. The Discussion should also contain an exploration of the temporal overlap between the gene expression effects (Fig1) and those on cell viability (Fig2), addressing the broader question of how gene expression can increase so strongly when cell death is so prevalent.

Minor comments:

- Please provide full information on the Methods employed in this study, especially cell culture and qPCR, rather than expecting readers to navigate to previously-published work.

- Did the qPCR analysis include technical replicates?

- For the calcein AM image analysis, specifically which regions were taken as background?

- '[Author University]' needs replacing on line 74!
